# A New Editor in Chief for Molecules (It’s Been a Fun Ride)

**DOI:** 10.3390/molecules25225423

**Published:** 2020-11-19

**Authors:** Derek McPhee

**Affiliations:** MDPI AG, St. Alban-Anlage 66, 4052 Basel, Switzerland; mcphee@mdpi.com

It is with mixed feelings that I put fingers to keyboard to write this, perhaps my last Editorial as Editor-in-Chief of *Molecules*. On the one hand, I have now been involved with *Molecules* in one way or another since 1998, so it has been a feature of over 2/3 of my professional life. On the other hand, as I retire from the position—effective the end of the year—I am extremely pleased that MDPI has access to an outstanding superb pool of candidates and Prof. Farid Chemat (Green Extraction Team, Avignon University, INRAE, Avignon Cedex 84029, France), the current Green Chemistry Section Editor-in-Chief of *Molecules*, has kindly accepted MDPI’s offer to replace me, effective Jan 2021. I have no doubts whatsoever that under his guidance and with the help of the journal’s excellent editorial staff in the MDPI offices around the world, *Molecules* will go on to even more and better accomplishments. 

Little could I imagine when in 1998, I answered an online ad from Dr Shu Kun Lin, the visionary founder of *Molecules* and MDPI, seeking someone to help the then Editor-in-Chief of *Molecules*, Dr. Esteban Pombo-Villar, run what was one of the first ever online chemistry journals. Intrigued by this new technology, I responded and was soon working in my spare time as editorial assistant, a fancy name for a one person editorial office handling manuscript receipt, peer review layout and editing, while the founding editor handled the publishing and administrative tasks, uploading the journal to the server of the University of Basel which had kindly donated some network space to host it. That first year we published 74 papers and the Molbank section of *Molecules* was introduced, publishing one-compound-per-paper short notes. That section (a predecessor of the 28 or so current and past sections that now make up *Molecules*) would eventually become an independent journal with its own ISSN (1422-8599) in 2003. The first annual ECSOC e-conference (http://www.mdpi.org/ecsoc/) was also held this year. 

Those first few years I would also come to be the editorial assistant for other MDPI journals as they were launched (*IJMS* and *Entropy* in 1999, *Sensors* in 2001 and *Marine Drugs* in 2003). Dr. Shu-Kun Lin took over as Editor-in-Chief of *Molecules*, replacing Dr. Pombo-Villar whose senior management duties with Novartis no longer allowed him to dedicate the necessary attention to the growing journal and I was formally appointed its Managing Editor, a position I would hold during the expansion of MDPI and the founding of the first formal MDPI editorial office to handle manuscripts in 2002. In 2005, the year *Molecules* celebrated its 10th anniversary, I was honored to be offered and accept the role of Editor-in-Chief.

I would be remiss if I did not mention here our dear colleague Dr. Francis Muguet (1955–2009), who joined MDPI in 2001 as an Associate Editor for *Molecules*, *Entropy* and *IJMS* and initiated production of the now collector item print versions of these three journals using the facilities kindly provided by his then employer, L’École Nationale Supérieure De Techniques Avancées (ENSTA) in Paris. Francis also ran several mirrors of the MDPI website (some still running today). A true Renaissance man (in addition to his PhD in Theoretical Chemistry he held a law degree that would serve him well in his other activities), Francis was an fervent and tireless defender of the concept of free and open access to scientific content, chairing the Civil Society Scientific Information Working Group, and coordinating the patents, trademarks and copyrights workgroup at the United Nations World Summit on the Information Society in 2003. He also designed the global patronage pattern to answer the legal arguments raised against the global license under the Berne Convention. The last few years until his untimely and sudden death he was a consultant to the International Telecommunication Union (ITU) and the University of Geneva. His contributions to the modern scientific information world we now take for granted cannot be overstated and he is sorely missed by all who knew him. 

There have been many milestones that I have had the pleasure of witnessing in the ensuing years. The introduction of the automated manuscript submission and manuscript handling system (SuSy) in 2011, the same year MDPI took its first baby steps on social media with a Facebook page. The first 100 employees in the various editorial offices in 2012 (a number that would reach 1000 five years later and now stands at just over 3500) and the iPhone and Android app versions of the MDPI platform made available via the iTunes and Google Play stores. Our 6000th paper, published in 2014. The 10,000th one only two years later. The first Tu Youyou Award announced in 2016, our most prestigious prize, that joined our Best Paper and Travel Awards. The journal’s highest Impact Factor (3.098) for the year 2017 (the 5-year impact factor is 3.268). Our move to semi-monthly issues in 2019 to handle the sheer volume of papers being submitted and published, and finally, our 20000th paper published earlier this year (from a total of 420,000 published by MDPI across all its journal portfolio). 

While it may sound like a cliché, as I hand over the pleasure and honor of being Editor-in-Chief of *Molecules* to a new colleague, I can truthfully state what I will miss most are the daily interactions with all the people involved, from my dear friend and colleague Dr. Shu Kun Lin, the founder of *Molecules* and MDPI, to our many authors and peer reviewers, readers and the *Molecules* Section Editors, the Managing Editors and Editorial Assistants from the MDPI offices around the globe with whom I have the honor of interacting all these years. I know I leave the journal in their very capable hands, and I can truly look back with admiration at our progress and say it has been a fun ride.

                    Derek McPhee                    Editor in Chief, *Molecules*

